# Mindfulness Enhances Episodic Memory Performance: Evidence from a Multimethod Investigation

**DOI:** 10.1371/journal.pone.0153309

**Published:** 2016-04-26

**Authors:** Kirk Warren Brown, Robert J. Goodman, Richard M. Ryan, Bhikkhu Anālayo

**Affiliations:** 1 Department of Psychology, Virginia Commonwealth University, 806 W. Franklin St., Richmond, VA, 23284, United States of America; 2 Institute of Positive Psychology and Education, Australian Catholic University, Locked Bag 2002, Strathfield, NSW, Australia 2135; 3 Clinical and Social Sciences in Psychology, Meliora Hall, P.O. Box 270266, University of Rochester, Rochester, NY 14627, United States of America; 4 Numata Center for Buddhist Studies, University of Hamburg, Alsterterrasse 1, 20354 Hamburg, Germany; 5 Kultur Indiens und Tibets, University of Hamburg, Alsterterrasse 1, 20354 Hamburg, Germany; University of California, San Francisco, UNITED STATES

## Abstract

Training in mindfulness, classically described as a receptive attentiveness to present events and experiences, has been shown to improve attention and working memory. Both are key to long-term memory formation, and the present three-study series used multiple methods to examine whether mindfulness would enhance episodic memory, a key form of long-term memory. In Study 1 (*N* = 143), a self-reported state of mindful attention predicted better recognition performance in the Remember-Know (R-K) paradigm. In Study 2 (*N* = 93), very brief training in a focused attention form of mindfulness also produced better recognition memory performance on the R-K task relative to a randomized, well-matched active control condition. Study 3 (*N* = 57) extended these findings by showing that relative to randomized active and inactive control conditions the effect of very brief mindfulness training generalized to free-recall memory performance. This study also found evidence for mediation of the mindfulness training—episodic memory relation by intrinsic motivation. These findings indicate that mindful attention can beneficially impact motivation and episodic memory, with potential implications for educational and occupational performance.

## Introduction

Mental time travel is a hallmark of human subjectivity, a key feature of which is the capacity to consciously re-experience past events. This *episodic memory*, the remembrance of past episodes from specific times and places, allows humans to guide their present and anticipated future behavior. By influencing what information is placed in working memory, episodic memory allows us to apply knowledge about our past to execute current tasks and to reflect on, plan for, and derive preferences for future situations [1]. A form of long-term memory, and specifically declarative memory (the other form being semantic memory), episodic memory is subject to both neurological insult and age-related decline. Given its importance, there is considerable demand for interventions to preserve and enhance episodic and other forms of memory [2].

The formation of episodic memories depends on encoding, the receipt and registering of sensory information, and is the first step in the creation of representations of experienced events or situations; these representations provide inputs to working memory and then, potentially, long-term memory, to be later retrieved as episodic and other types of memories. Early work in this area [3] demonstrated that controlled processing at encoding supports later retrieval. Convergently, when attentional capacities are taxed during encoding, the ability to recall those memories is hindered [4]. More recently, Bissig and Lustig [5] showed that the encoding stage could be an effective locus of memory training (see [6] for review), Indeed, considerable intervention research has targeted encoding processes, but to date evidence supporting the role of encoding-focused behavioral interventions to improve episodic memory in healthy adults is mixed [2].

Encoding is dependent on the degree or quality of attention paid to events and situations, and in everyday life can be easily compromised by distraction, mind-wandering, social cognition biases, and other perceptual interferences. The ability to control attention has been regarded as a core cognitive faculty [7–9] and simply put, paying close attention facilitates better memory of the stimuli attended to [10,11]. In recent years, interest has grown in the cognitive benefits of a form of attention called *mindfulness*, and incipient research indicates that it can improve attention and working memory. These findings suggest that mindful attention may have “downstream” effects on episodic memory as well [11], and the present research tested this proposition.

The concept of mindfulness has multiple meanings [12–14], but classical accounts relate a receptive attentiveness of present events and experiences to memory [15]. Usage of the term mindfulness in early Buddhist canonical texts makes explicit the link between mindfulness and episodic memory. Mindfulness is described as a capacity that facilitates the “ability to call to mind things that were done and said long ago” [16]. This aspect of the classical description of mindfulness in early Buddhist sources was the starting point of the present research, which took into account the historical roots in the Buddhist traditions of modern day mindfulness research.

Training in mindfulness takes two general forms termed “focused attention” and “open monitoring.” Training in focused attention (FA), the primary interest of the present studies, is often the first form that trainees undertake, and involves experiential practice in directing attention to chosen perceptual stimuli. FA training involves several attentional faculties: sustained attention to a target stimulus, disengagement from distracting perceptual stimuli (attention switching), and redirection of attention to the chosen stimulus (selective attention) [17]. Consistent with this, FA training to enhance mindfulness has been associated with better performance on both an attention orienting task [18] and sustained attention tasks [19–21]. FA training has also been shown to enhance working memory performance with verbal and non-verbal materials [22–24]. Neuroimaging research is consistent with these findings; Tang and colleagues [25,26] found mindfulness-integrated training-related changes in anterior cingulate cortex (ACC) connectivity, and in other frontal areas implicated in executive attention and working memory tasks [27–31]. Such effects are also consistent with our theoretical understanding of mindfulness, in that attention, particularly the sustained attention cultivated in mindfulness training, may make stimulus representations in working memory more stable and less subject to decay [11].

Given these influences on attention and working memory, mindfulness training may also impact long-term episodic memory. Attention is required to encode items into working memory, and computational memory models [32] suggest that encoded items sit in a working memory buffer in the process of being encoded into long-term episodic memory. Thus, if items are encoded with more fidelity into working memory, they may also be encoded into episodic memory with more detail (see [11] for review). To date very little research has examined the effect of mindfulness training on episodic memory. A recent quasi-experimental study [33] showed that relative to matched controls, mindfulness practitioners showed better verbal free recall of words from a list; but the form of training (FA or OM) was not controlled and the task represents a coarse measure of episodic memory [11]. Also suggestive is quasi-experimental neuroimaging research that has linked mindfulness training with increased gray matter density in the hippocampus [34,35], a brain region implicated in episodic memory functioning [36,37].

### Present research

The present three-study series was designed to examine the effect of mindfulness on episodic memory, indexed by two task outcomes. Study 1, a correlational study, explored the role of trait and state mindfulness in episodic memory performance in the Remember-Know (R-K) paradigm [38]. The R-K task is designed to dissociate the recognition of objects previously seen (“remember”; episodic memory) relative to felt familiarity of the object (“know”; semantic memory) and to mere guessing. The two forms of memory are related, in that recognition relies on familiarity with the stimulus, an automatic process, to access encoded information, but episodic memory also involves recollection, a conscious retrieval process [6, 39]. Interest in this study was in remember performance on the R-K task as an index of episodic memory.

Study 2 examined the effect of mindfulness on episodic memory experimentally, comparing the impact of brief FA mindfulness training on recognition memory in the R-K task relative to a structurally equivalent active control training. Based on prior research on mindfulness and working memory, we hypothesized that trait and/or state mindfulness (Study 1) and briefly trained mindfulness (Study 2) would conduce to better remembering (episodic memory) on the R-K task.

Study 3 sought to extend this inquiry in two ways. First, we examined the effect of FA-based mindfulness training relative to active and passive control conditions on a different index of episodic memory, namely free recall, which also involves consciously controlled memory retrieval. The decision to examine a second index of episodic memory was guided by the importance of demonstrating a generalizable beneficial effect of mindfulness. Ranganath et al. [2] have argued that the use of multiple tasks assessing the same process but having different rules, stimuli, and response modalities can increase confidence in the generalizability of training effects to novel contexts. In accord with the above-stated hypothesis, we expected that brief mindfulness training would enhance free recall performance relative to that observed in the control conditions.

The second extension provided by Study 3 was an effort to explain why mindfulness enhances episodic memory. We reasoned that the anticipated memory-enhancement afforded by mindful attention may be due, at least in part, to the association of mindfulness with *intrinsic motivation*–an innate tendency toward engagement in interesting and enjoyable activities [40]. Initial correlational research suggests that mindfulness may promote intrinsically motivated and related forms of autonomous, or self-determined functioning. For example, Brown and colleagues [41,42] found that dispositional mindfulness predicted greater reported autonomy in day-to-day activities among college students and working adults, as measured through experience sampling. Because autonomy is an essential basis for intrinsically motivated activity [43] these studies are suggestive of the positive influence mindfulness might have on intrinsically motivated behavior and, perhaps, the salutary consequences of such behavior for memory task performance. Indeed, intrinsic motivation has been shown to enhance episodic (free recall) memory performance [44]. Therefore, we explored whether mindfulness would enhance free recall performance both directly, and through its association with enhanced intrinsic motivation.

If as hypothesized mindfulness does enhance episodic memory performance, the findings of the present research may have implications for improving educational and occupational performance, both of which rely on capacities to recognize and recall stimuli encoded in the past. Since motivation is a key factor in such settings, this research may also inform about a key process through which mindfulness training can better enhance episodic memory performance.

## Study 1

A correlational study was first undertaken to test the role of trait and state mindfulness in episodic memory performance. Theorized to represent individual differences in the frequency of day-to-day states of mindfulness, several self-report measures of basic trait mindfulness have been associated with such cognitive features as attention and working memory [45]. In this study, participants completed two measures of trait mindfulness emphasizing present-centered attention before completing the R-K task. Because trait mindfulness represents a general tendency to “be present” and so may not inform about quality of attention upon entry into the episodic memory task, self-reported state, or current mindfulness was also assessed immediately prior to the R-K task. In addition, we tested the potential role of trait *attentional control* on episodic memory performance. There is some debate about whether trait measures of mindfulness actually assess this subtle construct rather than related phenomena such as concentration [46]. In accord with theory and research on mindfulness measures [47] we anticipated that higher trait and/or state mindfulness, but not attentional control, would predict higher remembering relative to familiarity (knowing) and guessing.

### Materials and Methods

#### Participants

Participants were 144 introductory psychology students at a large mid-Atlantic university who provided written consent to participate and earned research participation course credit. One participant was excluded from analyses due to careless responding as indicated by three or more incorrect responses to directed questions [48]. The remaining 143 participants (n = 90 (63.4%) female) ranged in age from 18 to 37 years (*M* = 20.08, *SD* = 2.95). Most (42.3%) were Caucasian; the remainder were Asian (18.3%), African-American (17.6%), Hispanic/Latino (10.6%), or another race or ethnicity (11.2%). The Institutional Review Board at Virginia Commonwealth University approved the consent and other study procedures.

#### Procedure

Sessions were conducted with 1–7 participants in the same room, each seated in a cubicle separated with privacy dividers. Using MediaLab 2008 software (Empirisoft, New York, NY), participants first completed self-report trait measures of mindful attention (as well as dispositional tendencies toward memory errors and cognitive failures; data not reported here). Upon individual completion of these measures, the experimenter provided each waiting participant with a paper word search puzzle that served as a filler task until all participants in the session had completed the self-report measures. Once all participants had done so, the experimenter collected the word puzzles and delivered detailed instructions on the learning phase of the R-K task (see *Measures* below), which was followed by a short series of practice trials. They then completed a brief self-report measure of state mindful attention, and immediately began the R-K task. Once finished, each participant completed a demographics survey, was debriefed, and then dismissed.

#### Measures

Mindfulness. Two widely used measures tapped individual differences in a basic form of mindfulness, namely the Mindful Attention Awareness Scale (MAAS [41]; sample *α* = .82) and the Act with Awareness Subscale of the Five Factor Mindfulness Questionnaire (FFMQ-AW [49]; sample *α* = .90). The 15-item MAAS assesses the day-to-day frequency with which people are receptively attentive to and aware of current, or present moment events and experiences. Responses are made on a 6-point Likert scale (*almost always* to *almost never*) to items such as "I find it difficult to stay focused on what’s happening in the present." The 8-item FFMQ-AW similarly assesses individual differences in the tendency to be actively attentive to current stimuli using a 1 (*never or very rarely true*) to 5 (*very true or always true*) Likert scale. Higher scores on the MAAS and FFMQ-AW indicate higher levels of basic trait mindfulness.

State mindfulness was assessed using the 5-item state variant of the MAAS [41] (sample *α* = .85), which taps receptive attentiveness to present stimuli at a given point in time. Items are rated on a 7-point Likert-type scale anchored at 0 (*not at all*), 3 (*somewhat*) and 6 (*very much*). After reverse scoring each item and averaging the responses, higher scores reflected higher state mindfulness.

Attentional Control. The 20-item *Attentional Control Scale* (ACS; [50]) measured two major trait components of attention (focusing and shifting) on a 4-point scale (*almost never* to *always*). Higher scores indicate higher attention control (α = .71).

Recognition Memory Task. To assess the accuracy of recognition memory relative to other, dissociable types of recognition experience, participants completed the *Remember-Know task* (R-K; [38, 51]). The task consists of a learning phase and a test phase. During the learning phase, participants passively viewed a sequence of 60 randomly presented, colored images of easily recognizable everyday objects [52]. Each image was presented centrally, on a white background, via computer monitor for a duration of 5 sec, and preceded by a black fixation cross presented centrally on the screen for a duration of 1 sec. The interstimulus interval was 1 sec. Participants were instructed to view each image as closely as possible to better remember them, and were informed they would be tested on their memory for all presented objects.

After the learning phase, instructions on the test (memory) phase of the task were presented on the computer screen as well as given verbally by the experimenter. These instructions included a description of how to appropriately use the “old”, “new”, “remember”, “know”, and “guess” response options, as described in previous studies [53,54]. Participants were instructed to respond “O” (old) if they recognized that the object was presented during the learning phase, and to respond “N” (new) if the object was not presented during the learning phase. The O and N response options appeared below each image until a button box response was made. Half of the 120 test phase images were old and half were new; they were presented in random order, preceded by a 1 sec black fixation cross, and presented centrally for 2 sec. When an N response was made, a 1 sec intertrial interval followed. If an “O” response was made, a screen prompt was given to characterize the recognition experience as “remember”, “know”, or “guess,” in which “R” (remember) indicated “a vivid recollection of their past experience viewing the picture”; “K” (know) indicated “strong feelings of familiarity without vivid recollection”; and “G) (guess) indicated “guessing the picture was displayed before.” The descriptions of each option were presented verbally by the experimenter at pre-test phase training and were shown again on the computer monitor at the start of the test phase. The R, K, and G prompts appeared below each image until a button box response was made. Before starting the test phase, participants completed 10 practice trials and were given opportunity to ask questions of the experimenter.

A Signal Detection Theory approach was taken to effectively dissociate memory retention from decision-making processes during R, K, and G recognition judgments. Following the recommendations of Donaldson [55], hit rate and false alarm rate data were used to compute A*′* (A-prime) for R, K, and G. A*′* is a non-parametric index of discriminability (similar to *d′*), that accommodates hit or false alarm rates of 1 or 0. A′ values were computed as follows [56]:

$$A^{'}\;=\;.5+\left[sign(H-F)\frac{{\left(H-F\right)}^{2\;}+\;\left|H-F\right|}{4\;\text{max}\left(H,F\right)-4HF}\right]$$

Where sign(*H*-*F*) equals +1 if *H-F* > 0; 0 if *H* = *F*; and -1 otherwise; max(*H*, *F*) equals either *H* or *F*, whichever is greater. Higher A′ scores indicate more accurate discrimination between old and new items.

### Results and Discussion

#### R-K memory performance

R-K task performance results showing remember, know, and guess hit and false alarm rates is shown in Table 1. A mixed-measures ANOVA was conducted to assess differences in recognition accuracy between remember, know, and guess responses while controlling for the main and interactive roles of participant sex and trait attentional control. There was a main effect of recognition performance, *F*(2,204) = 92.39, *p* < .001, *η*_*p*_^2^ = .48. As in past research [51], planned contrasts indicated that remember responses were significantly more accurate than know responses, *F*(1,102) = 44.23, *p* < .001, *η*_*p*_^2^ = .30. Know responses were significantly more accurate than guess responses *F*(1,102) = 47.74, *p* < .001, *η*_*p*_^2^ = .32. A preliminary analysis showed no main effects of participant sex (*p* = .91) or trait attentional control (*p* = .97), nor any sex or trait attention control interactions with recognition performance (*p*s > .22). These two variables were therefore excluded from subsequent models.

**Table 1:** Mean (and Standard Error) Hit and False Alarm Rates in the Remember-Know Task (Study 1).

	Hits	False Alarms
Remember (R)	.61 (.02)	.01 (.002)
Know (K)	.20 (.02)	.02 (.003)
Guess (G)	.06 (.01)	.05 (.01)

*Notes*. *N* = 143. Hits represent the proportion of targets correctly endorsed as old; false alarms represent the proportion of lures (new images) endorsed as old.

#### Mindfulness and R-K episodic memory performance

The MAAS and FFMQ-AW trait mindfulness measures were highly correlated (*r* = .68, *p* < .001) but were treated separately in analyses because they contain 5 of the same items (the FFMQ-AW was derived, in part, from the MAAS) and are commonly treated as distinct measures in research. State MAAS scores were correlated with MAAS and FFMQ-AW trait scores (*r*s = .40 and .44, respectively, *p*s *<* .001). Correlations between measures of mindfulness, attentional control, and R-K memory performance are shown in Table 2. Three mixed ANOVA models were tested, each covarying one self-reported measure of mindfulness: trait MAAS, FFMQ, and state MAAS. Neither MAAS nor FFMQ trait mindfulness scores predicted A′ discriminability differences between remember and know, and know and guess responses (all *p*s > .68). However, in the model covarying state MAAS scores, there was a significant state MAAS × recognition performance interaction, *F*(2,284) = 4.89, *p* = .008, *η*_*p*_^2^ = .03. Simple effects tests indicated that higher state MAAS scores were associated with improved recognition performance specific to remember responses, *t*(141) = 4.50, *p* < .001, *η*_*p*_^2^ = .13. There was no associations between state MAAS and knowing (*p* = .16) or guessing (*p* = .24).

**Table 2:** Correlations between Measures of Mindfulness, Attentional Control, and Remember-Know Task Performance (Study 1).

R-K Memory Performance	ACS	MAAS	FFMQ-AW	S-MAAS
Remember (A*′*)	.06	.12	.09	.35***
Know (A*′*)	-.02	-.04	-.05	-.12
Guess (A*′*)	.08	-.04	.05	.10

*Notes*. *N* = 143. A*′* values represent recognition accuracy of target stimuli. ACS = Attention Control Scale; MAAS = Mindful Attention Awareness Scale; FFMQ-AW = Five-Factor Mindfulness Questionnaire Act with Awareness Subscale; S-MAAS = State Mindful Attention Awareness Scale.

*** *p* < .001

Thus trait mindfulness did not predict episodic memory performance, but higher state mindfulness immediately prior to the task predicted the ability to differentially remember old stimuli relative to new stimuli (better episodic memory) but not better semantic memory (feeling familiar with stimuli) or guessing. This study therefore provided initial evidence consistent with our hypothesis that mindful attention promotes better episodic memory performance.

## Study 2

Based on the hypothesis-consistent findings of Study 1, a second study was undertaken using an experimental design to better examine the causal role of mindfulness in episodic memory performance. The effect on episodic memory of very brief instruction in mindfulness emphasizing FA was tested (c.f., [57–59]), relative to a structurally equivalent active control instruction emphasizing attention to personal goals. Brief laboratory manipulations such as those used here have informed our understanding of information processing and its effects under controlled conditions [60,61]. Importantly, a very brief training in mindfulness is not designed “to analogize a full mindfulness treatment program, but to bring about very short term changes that would need much longer to consolidate if they were going to bring about long-term benefit” ([61] p. 3).

With this granted, longer-term mindfulness training is typically multi-model, involving didactic instruction, social support, and other non-specific factors in addition to multiple forms of mindfulness practice, leaving unclear which training ingredients are active in producing outcomes of interest, particularly in the absence of closely matched control conditions. Brief mindfulness trainings offer complementary value by permitting a close examination of specific forms of mindfulness training—FA in the present case—that can inform about cognitive (and other) effects of mindful states quite directly. Finally, this study tested an alternative hypothesis that training condition differences in episodic memory may be due to different levels of attention to and engagement with the training instructions, and relatedly, baseline participant energy, or fatigue.

### Materials and Methods

#### Participants

Participants were 99 introductory psychology students at a large mid-Atlantic university who earned course credit for participation. Two participants were excluded from analyses due to careless responding as indicated by three or more incorrect responses to directed questions [48]. Four participants were excluded from analyses for inattentiveness or noncompliance during the training phase (e.g., looking around the room when instructed to close their eyes; removing headphones before the end of the training). The remaining sample of 93 participants (n = 66 (71%) female; 2 undeclared) ranged in age from 18 to 27 years (*M* = 19.12, *SD* = 1.86). Most (38.7%) were Caucasian; the remainder were African American (19.4%), Asian (15.1%), Hispanic/Latino (12.9%), or another race or ethnicity (13.9%). Due to a procedural error, two participants did not complete the concluding manipulation check items. The Institutional Review Board at Virginia Commonwealth University approved the consent and other study procedures.

#### Procedure

Participants were tested in groups of 1–7, with each participant seated in the same room at a cubicle separated with privacy dividers. All participants were given the cover story that the research was exploring “how active or engaged learning styles promote better academic outcomes.” Then participants completed a baseline self-report measure of state (current) fatigue (as well as trait attentional control; data not reported here) to permit statistical control for baseline energy level (see *Measures* below).

Participants were then randomly assigned to an FA form of mindfulness training (*n* = 44) or a structurally equivalent control training condition (*n* = 49). Both conditions involved listening through headphones to two brief audio recordings (9 min, 40 sec)–once before the learning phase of the memory task, and once before the test phase of the task. Each recording consisted of condition-specific instructions delivered by a male speaker and a female speaker. The use of both male- and female-delivered instructions in each condition was designed to insure that instructional effects on the R-K outcomes were not confounded with speaker sex. The order of the recordings in each condition was randomized, such that half of the participants received the male speaker before the learning phase, and half received the male speaker before the test phase. To facilitate engagement during the audio recordings, all participants were told they had all been assigned to an “active engagement” condition, while “participants in other experimental sessions had been assigned to a “distraction” condition (see S1 Text for training instructions).

The male-voice mindfulness training was derived, with slight adaptation, from an exercise in [62]. The female-voice recorded mindfulness instruction was adapted from [63]. Both provided guided instruction to promote a state of receptive attention to present-moment sensations in the body, and particularly to the physical sensations associated with breathing. The recordings also instructed the listener to briefly mentally note the occurrence of thoughts and emotions any time they noticed their mind had wandered from attending to the breath, and to gently regather their attention to focus on the physical sensations associated with breathing.

The audio recordings in the control condition were identical in speaker sex and duration to those in the mindfulness condition, and were counterbalanced in the same way. The training delivered by a male speaker instructed on the importance of “putting first things first” when planning for the future, one of Covey’s [64] “seven habits of highly effective people.” The instructions asked participants to visualize aspects of their life that were important to them, and explained how to incorporate these important aspects when planning for the future. The female-delivered audio instructions involved “the way humans think and perceive reality,” covering the complexities of the brain and how humans are similar to and different from other animals [65].

Immediately following the first audio recording, participants began the learning phase of the R-K task, which was identical to that in Study 1. Then, as in Study 1, before the test phase participants received verbal and computer-mediated instructions on the distinction between “remember”, “know”, and “guess” responses, and completed 10 practice trials. After this all participants completed a second 9-min, 40 sec audio training consistent with their randomly assigned condition. The test phase of the R-K task was then completed using the same parameters as in Study 1. Upon completion, each participant completed a short series of manipulation check items to assess their experiences while listening to the audio recordings (see *Measures* below). They were then debriefed and dismissed.

#### Measures

Fatigue. The 7-item Profile of Mood States (POMS [66]; α = .91) fatigue subscale was administered using a 1 (*not at all*) *to* 5 (*extremely*) Likert scale. Participants were instructed to respond based on how they felt “right now.” Higher scores indicated a higher level of fatigue.

Training manipulation check. To determine whether participants in the two conditions were equally engaged with the recordings, they responded to 5 items about their experience while listening to the audio recordings immediately after the test phase of the R-K task (e.g., “how *concentrated* did you feel while listening to the audio recordings?”) using a 1 (*very slightly or not at all*) to 5 (*extremely*) Likert scale. These items, adapted from the Positive Affect Negative Affect Schedule—Expanded form (PANAS-X [67]) were used to assess states associated with attentiveness (*attentive*, *concentrated;* sample α = .85) and task engagement (*bored* (r), *disconnected* (r), *and interested;* sample α = .64).

### Results and Discussion

Participants in the mindfulness condition did not differ in self-reported attentiveness (*M* = 2.93, *SD* = 1.02) from those in the control condition (*M* = 2.84, *SD* = .98) during the audio instructions, *t* (89) = -.41, *p* = .68, *d* = -.09. Likewise the former condition (*M* = 3.00, *SD* = .93) did not differ from the latter condition (*M* = 2.86, *SD* = .87) in degree of instructional task engagement, *t*(89) = -.70, *p* = .49, *d* = -.15.

Table 3 depicts the mean hit and false alarm rates of old/new decisions as a function of response category (remember, know, and guess). Hit and false alarm rate data were used to compute A′ measures of stimulus discriminability using the identical procedure in Study 1. A preliminary mixed-model ANOVA was conducted to assess differences in recognition accuracy between remember, know, and guess responses while controlling for the main and interactive roles of participant sex, baseline fatigue, and instruction speaker order (male first *vs* female first). There was no main or interactive effect of fatigue or instruction speaker order on recognition performance (*p*s > .26 and *p*s > .09, respectively). A main effect for participant sex on recognition performance was found (*F*(1,87) = 4.53, p = .04, *η*_*p*_^2^ = .05), such that females’ old/new stimulus discrimination was more accurate across remember, know, and guess categories. However, participant sex did not interact with recognition performance (*p* = .16).

**Table 3:** Mean (and Standard Error) Hit and False Alarm Rates during the Remember-Know Task (Study 2).

Response Category	Hits	False Alarms
Remember	.62 (.03)	.02 (.003)
Know	.19 (.02)	.02 (.003)
Guess	.05 (.01)	.05 (.01)

*Notes*. *N* = 93. Hits represent the proportion of targets endorsed as old; false alarms represent the proportion of lures endorsed as old.

#### Brief mindfulness training and R-K episodic memory performance

To assess the effect of brief mindfulness training on recognition performance, a mixed model ANOVA was conducted while controlling for participant sex. As expected, there was a significant main effect of recognition performance, *F*(2,176) = 88.57, *p* < .001, *η*_*p*_^2^ = .50. Consistent with Study 1, planned comparisons indicated that remember performance was significantly more accurate than for knowing, *F*(1,88) = 34.44, *p* < .001, *η*_*p*_^2^ = .28. Recognition performance for knowing was also greater than for guessing, *F*(1,88) = 59.17, *p* < .001, *η*_*p*_^2^ = .40. There was no main effect for training condition (*p* = .77). Importantly, there was a significant training condition × recognition performance interaction, *F*(2, 176) = 3.70, *p* = .03, *η*_*p*_^2^ = .04. Those in the mindfulness condition (*M* = .91, *SD* = .09) were significantly more accurate than controls (*M* = .85, *SD* = .13) in discriminating old from new stimuli when their recognition was characterized by remembering, *F*(1,88) = 7.61, *p* = .007, *η*_*p*_^2^ = .08 (see Table 4). There was no significant training condition difference between knowing and guessing (*p* = .26).

**Table 4:** Mean (and Standard Error) A*′* Values in the Remember-Know Task by Experimental Condition (Study 2).

A*′*	Mindfulness	Control	*F*	*p*	*η*_*p*_^2^
Remember	.91 (.02)	.84 (.02)	7.61	.007	.08
Know	.70 (.02)	.73 (.02)	1.21	.28	.01
Guess	.51 (.02)	.55 (.02)	.97	.33	.31

*Notes*. *N* = 93. A*′* values represent recognition accuracy of target stimuli.

Consistent with and extending the results of Study 1, this study provided experimental support for our hypothesis that mindful attention can promote episodic memory performance. One limitation of this study concerns the inability to know whether instructional speaker sex had some effect on the R-K outcomes. Since these outcomes were collected after participants listened to both instructional recordings, we cannot determine whether male and female speakers would have similar effects on these outcomes even though, as noted earlier, order of speaker (male or female recording first) had no effect on the outcomes. An additional limitation concerns the fact that content of the two instructional sets in the control condition differed, but did not differ substantially in the mindfulness condition. To examine whether this had some effect on the R-K outcomes, terms representing the instructional (speaker) order × experimental condition interaction, and instructional (speaker) order × experimental condition × recognition performance interaction were added to the main model just reported. This analysis showed that neither interaction was significant (*p*s = .18 and .17, respectively), indicating that instructional content had no apparent effect on R-K task performance.

## Study 3

A third study was undertaken with two purposes in mind. First we sought to examine whether brief FA mindfulness training, relative to both normative and distracted states of mind, would lead to better performance on a different index of episodic memory than the recognition memory tested in Studies 1 and 2 –namely free recall of reading material. Of course reading, and the retention of material read, is central to knowledge acquisition through education, and to performance in a wide variety of occupations; thus memory of textual material represents an important outcome. In this study’s design, participants listened to one instructional recording only, and the same male speaker-delivered mindfulness training as in Study 2 was tested. Two control conditions were included: a distraction condition, in which the FA instruction was partially masked by white noise, served to model sonic conditions commonly present in modern urban environments. A no-instruction condition served to test that FA training in fact increased memory performance, rather than a control instruction decreasing it. In accord with our primary hypothesis, we predicted that a state of mindful attention, relative to the control condition states, would lead to better free recall memory of the text read.

The second purpose of this study was to explore whether mindfulness would have at least some of its effect on this memory performance through (that is, mediated by) heightened intrinsic motivation, as measured by greater reading task-specific interest and enjoyment. Intrinsic motivation is theorized to be an important process through which attentional engagement enhances cognitive and other performance [68]. In addition, given prior linkages between mindfulness and autonomous motivation, including intrinsic motivation [41,42], and intrinsic motivation and free recall [44], there was reason to believe that intrinsic motivation may mediate the relation between mindful attention and episodic memory.

### Materials and Methods

#### Participants

Fifty-seven undergraduates (72% female) at a Northeastern U.S. university provided written consent to participate and earned research participation course credit (due to a procedural error, other demographic data were not collected). The sole inclusion criterion was ability to read and write in English. The Research Subjects Review Board at the University of Rochester approved Consent and other study procedures were approved by the University’s Research Subjects Review Board.

#### Procedure

Participants were tested individually, each in the same laboratory room. They first completed a measure of current fatigue to permit statistical control of baseline energy level on the study outcomes as needed (see *Measures* below). They then read a 375-word factual text passage on the May Day holiday. After the reading, participants completed a self-report measure of intrinsic motivation (interest, enjoyment) and, to supplement the assessment of task enjoyment, a self-report measure of positive and negative affect (see *Measures* below). The reading, and the measures that followed, served to establish baseline, task-specific motivation and affective state.

Participants were then individually randomized to the mindfulness condition, a distraction control condition, or a no-task (waiting) control condition (*n* = 19 per experimental condition) and told that the purpose of this phase was to “make sure everyone is in the same mental state.” In the mindfulness condition, participants listened through headphones to an audio cassette-taped version of the male-voice recording used in Study 2 [62].

The distraction control condition was identical except that the instructional audiotape included continuous background white noise, consistent with prior distraction research [69], along with brief, random bursts of higher decibel noise during both the speech and silent portions of the instructions (median burst = 3.25 sec; total time = 2 min, 25 sec). This noise combination was designed to mimic common sonic features of modern environments [70], in which there is often continuous background noise (e.g., heating/cooling systems, traffic) punctuated at random by brief bursts of higher decibel noise (e.g., car horns, sirens). The signal-continuous noise difference was 22.6 decibels (db), favoring signal; with noise bursts the difference ranged from 10.3 to 6.9 db, favoring signal. While no noise was introduced into the mindfulness instruction audio, normative background tape noise was measured; the signal-noise difference was 30 db. Shifting of attention from the primary, instruction task to irrelevant noise is a potential concern in this approach, but the likelihood of this was minimized by the non-semantic nature of the noise and by removing any demand to attend to noise or to report on any of its features; instead, interest was in whether the noise would disrupt the processing of task instructions [71]. To justify the noise, participants were told that the tape quality was poor. This condition served to control for attention to audio-based instructions. In the wait/no-task control condition, equal in duration to the other conditions, participants were instructed though headphones to wait without engaging in other activities. This condition served to control for passage of time between readings and measures completion (see S1 Text for training instructions).

Participants then read a second, 372-word biographical text passage on the author Rudyard Kipling (c.f., [44]). This passage and the May Day passage used in baseline assessment were rated as moderately interesting, on average, in pilot testing; this allowed for maximal within-person increases or decreases in motivation as a result of the attention manipulations described earlier. After the second reading, participants completed the same measures of motivation and affect as before. Participants were then asked to sit and relax while refraining from other activities until the next part of the study was prepared. This 4-min period of inactivity introduced a time lag between the second text reading and the free recall of it, while also minimizing interference from other activities. The participant was then given a blank page and instructed to “write everything you remember from the second passage you just read, including as much detail as possible. Please write any titles, sentences, ideas, or facts you recall.” After exactly 5 minutes, the experimenter returned and asked the participant to stop writing. A manipulation check followed, in which two questions were asked: “How easy was it for you to follow the instructions on the tape?” (7-point scale; *very difficult* to *very easy*) and “To what extent were you able to concentrate on the instructions on the tape?” (7-point scale; *not at all* to *extremely*). We then asked each participant whether anyone had told them about the study before their session; all indicated ‘No.’ After probes for suspicion and awareness of the study purpose, the participant was debriefed, thanked, and dismissed.

#### Measures

Fatigue. The 7-item Profile of Mood States (POMS) fatigue subscale [66] assessed baseline fatigue “right now” on a 5-point scale (*not at all* to *extremely*) (α = .92).

Intrinsic motivation. The 5-item interest/enjoyment subscale of the Intrinsic Motivation Inventory (IMI; [72]) measured enjoyment and interest in the text readings on a 7-point scale (*not at all true* to *very true*), as measured following each reading. An example statement is, “I would describe this material as very interesting” (baseline α = .91).

Affect. The 20-item Positive and Negative Affect Schedule (PANAS [73]) assessed affective states on a 7-point scale (*not at all* to *extremely*) immediately after each reading with the instructions, “During the reading of this passage, to what degree did you experience the following emotions?” (baseline PA α = .89; baseline NA α = .77).

Free recall memory. Two trained assistants, naïve to the study hypotheses and participants’ experimental condition, independently coded participants’ free recall text for propositions, where a proposition was defined as a predictor and arguments, combined by specified rules [74,75] (see [44] for coding details). Inter-rater reliability was high (intraclass correlation = .98). Differences were resolved by discussion between the two raters.

### Results and Discussion

Table 5 (top portion) displays descriptive statistics on the baseline (pre-manipulation) study variables according to experimental condition. Preliminary Analyses of Variance (ANOVA) found no differences between conditions in baseline PA (*p =* .73; η_p_^2^ = .01), NA (*p =* .41; η_p_^2^ = .03), nor IMI intrinsic motivation (*p =* .06; η_p_^2^ = .10). However, given their importance these baseline variables were controlled in relevant primary analyses.

**Table 5:** Descriptive Statistics on Baseline and Post-task Variables by Experimental Condition (Study 3).

	Mindfulness			Distraction			Wait/No task		
*Variable*	*M*	*SD*	*Range*	*M*	*SD*	*Range*	*M*	*SD*	*Range*
*Baseline*									
Intrinsic motivation	3.45	1.06	1.80–5.40	3.00	1.23	1.00–5.40	3.96	1.30	1.40–6.00
Positive affect	2.70	0.99	1.30–4.90	2.88	1.00	1.10–4.90	2.62	1.11	1.40–4.90
Negative affect	1.28	0.40	1.00–2.60	1.37	0.33	1.00–2.10	1.25	0.34	1.00–2.30
*Post-task*									
Intrinsic motivation	3.91	1.42	1.60–6.80	2.73	1.37	1.00–5.80	3.18	0.91	1.80–5.00
Positive affect	2.82	1.29	1.10–5.10	2.47	1.04	1.00–4.50	2.18	0.86	1.30–3.90
Negative affect	1.13	0.35	1.00–2.44	1.59	0.75	1.00–4.10	1.27	0.28	1.00–1.70
Free recall memory	7.58	4.23	2.00–17.00	3.37	1.89	0.00–7.00	4.32	3.15	0.00–11.00

*Note*. *N* = 57 (*n* = 19 per condition).

Analyses of the manipulation check questions suggested that the experimental manipulation was effective, with participants in the mindfulness and no-task control conditions reporting more ease in following the audio instructions than those in the distraction condition (*p*s < .002; Cohen’s *d*s ≥ .92). Mindfulness participants also reported higher ability to concentrate on the instructions than distraction participants (*p =* .002; *d* = .98). Neither participant sex (all ps > .10) nor baseline POMS fatigue (all ps > .17) were significant predictors of the four outcomes, so were not further considered.

#### Primary analyses

Table 5 (bottom portion) presents descriptive statistics on the post-task (post-instruction) outcomes of the study by condition. General linear models, specifically Analyses of Covariance (ANCOVA) tested the effect of task condition on these outcomes while covarying the respective baseline scores. In the model predicting IMI motivation, task condition was a significant predictor (F(2,53) = 3.95, *p =* .03; η_p_^2^ = .13) while baseline IMI score was not (F(1,53) = 1.36, *p =* .25; η_p_^2^ = .03). As hypothesized, post-hoc t-tests showed that post-task IMI scores were higher in the mindfulness condition than in the distraction condition (*p =* .01; *d* = .84) but contrary to our hypothesis, these scores were not higher in the mindfulness condition than in the no-task control condition (*p =* .06; *d* = .61). The two control conditions did not differ from each other in post-task IMI score (*p =* .50; *d* = .39).

To further test the hypothesized role of the mindfulness condition in promoting greater task enjoyment, we examined task condition effects on the positive and negative affective state outcomes. In the prediction of post-task PA, baseline PA was a significant predictor (F(1,53) = 36.23, p < .0001; η_p_^2^ = .41) while task condition was not (F(2,53) = 2.64, *p =* .08; η_p_^2^ = .09). In the prediction of post-task NA, task condition was a significant predictor (F(2,53) = 5.38, *p =* .008; η_p_^2^ = .17), as was baseline NA (F(1,53) = 9.16, *p =* .004; η_p_^2^ = .15). Post-hoc t-tests showed that post-task NA was lower in the mindfulness condition than in the distraction control condition (*p =* .002; *d* = 1.06) and in the no-task control condition (*p =* .04; *d* = .70). The two control conditions did not differ in NA (*p =* .24; *d* = .49).

In an ANOVA model of free recall memory performance, task condition was a significant predictor (F(2,54) = 8.86, *p =* .0005; η_p_^2^ = .25). As hypothesized, participants in the mindfulness condition showed better free recall performance, indicated by a higher number of propositions recalled than those in the distraction group (*p =* .0002; *d* = 1.28) and in the no-task group (*p =* .003; *d* = .88). The two control conditions did not differ in number of propositions recalled (*p =* .37; *d* = .37).

Finally, it is possible that the task condition results reported here were not due to the mindfulness instructions, but rather due to lack of interest or boredom, inattentiveness, or irritability in the distraction and/or no-task control conditions. To test these possibilities, ANCOVA models regressed post-task measures of these three states of mind, derived from three of the state PANAS items (*interested*, *attentive*, *irritable*), on task condition and baseline measures of the same variables. Scores on the three outcomes did not differ by task condition (all *p*s > .08; η_p_^2^ < .09). Baseline scores on these variables did predict the corresponding post-task scores (all *p*s < .02; η_p_^2^ > .10), indicating that boredom, inattentiveness, and irritability after the induction were related to pre-existing levels of these states rather than to task condition.

#### Mediation analyses

To examine whether intrinsic motivation mediated the relation between task condition and memory performance, a general linear regression model first regressed post-task IMI motivation on dummy-coded task condition (mindfulness versus both control conditions) while controlling for baseline IMI score. Similarly to the motivation results reported earlier, task condition predicted post-task IMI score, *p =* .008. In a second model, post-task IMI score predicted memory performance, *p =* .0002. Finally, we regressed memory performance on task condition and then both task condition and post-task intrinsic motivation. The task condition—memory performance relation dropped from *β* = .49, *p* < .0001 in the simple regression model to *β* = .37, *p =* .003 after entry of IMI score into the model. In this latter model, IMI score also predicted memory performance, *β* = .35, *p =* .004. To determine whether the partial mediation effect of motivation was significant, we used the *z′* test and the product test recommended by [76]. In both tests, intrinsic motivation significantly mediated the task condition—memory relation, *z′* = 2.26 and *P* = 10.92, respectively, both *p*s < .01.

## General Discussion

Episodic memory formation is dependent on sensory encoding and working memory processes, and researchers have sought to enhance episodic memory by improving initial encoding through various forms of training, but with mixed success [2]. Building upon scholarship on early Buddhist canonical descriptions of mindfulness [16] and prior empirical work showing that mindfulness training improves both attentional and working memory processes [11], we hypothesized that bringing mindful attention to bear on task stimuli would improve episodic memory. Supporting this hypothesis, a multimethod study series first showed that a self-reported state of mindful attention (but not self-reported trait mindfulness) predicted better recognition performance on the R-K task (Study 1). Two experiments then showed that very brief, focused attention-based mindfulness training improved R-K recognition performance (Study 2) and reading task-based free recall performance (Study 3). An exploration of mediation in Study 3 also showed that intrinsic motivation partially explained the effect of mindfulness training on free recall success. These findings, particularly in Studies 2 and 3 were associated with moderate effect sizes. Thus, using two operationalizations of mindfulness and two indexes of episodic memory, the study findings present a consistent picture of benefit from mindful attention in episodic memory enhancement.

More generally, these results support the proposition that training in sensory encoding is a suitable target for long-term memory enhancement [2]. Established theories of both memory [77] and intrinsic motivation [40,78] have indicated that task engagement and task performance are impacted by the quality of attention paid to the task. Since mindfulness training has been shown to enhance key attention capacities, including orienting and alerting [18], the increased moment-to-moment attention associated with mindful task engagement may directly contribute to enhanced working memory [11] and in turn the better episodic memory performance observed here. However, Study 3 found evidence for an indirect effect, in that mindfulness training led to stronger task engagement (higher intrinsic motivation and, supplementarily, lower negative affect), which partially mediated the training effect on episodic memory. Since it was examined in only one study with a comparatively small sample, this mediation finding requires replication before it can be considered reliable; but it is consistent with research showing trait mindfulness to predict higher day-to-day autonomous (including intrinsic) motivation and lower negative affect [41,42], and with research highlighting the beneficial effect of intrinsic motivation on episodic (free recall) memory [44]. Research is needed to examine all three steps of the memory process discussed here—encoding, working memory, and episodic memory—to better understand the role of mindfulness in memory formation and retention, as well as the processes, like intrinsic motivation, that help to explain how mindfulness enhances the success of these memory outcomes.

### Limitations and future research

Like much previous research in this area, these studies were conducted with university student samples, and tests of the generalizability of the results to other populations, such as older adults or less educated samples, are needed. Also, because we did not measure or otherwise control for participant expectancy effects on the outcomes, we cannot definitively conclude that expectancies did not bear some influence on the study results. There are reasons to believe that expectancy is not a likely alternative explanation for the condition differences found, including the fact that participants had no prior knowledge of the study hypotheses or procedures, and the audio instructions bore no resemblance to, nor referred to any elements of the task that followed, nor its outcomes. However, in future selecting active control interventions and outcome measures based on prior, independent assessments of expectations could help to rule out expectancy effects [79].

The mindfulness training findings are consistent with the claim that the task condition differences in motivation and memory were due to a mindfulness-related enhancement of experience and performance. Yet Study 2 did not include a no-instruction control condition, and so it is possible on methodological grounds alone that the active control condition training in attention to goals detrimentally impacted memory performance. The results of Study 3 inveigh against this interpretation, as performance in an active control condition (distraction) was nonsignificantly different from a no-instruction control condition. Future research could use active control conditions that provide a stronger test of the effect of mindfulness training on episodic memory, namely empirically validated training in attention to enhance encoding.

An additional direction for future research is to examine other points in the memory process at which mindful attention may have effects. The present studies focused on memory enhancement at the encoding stage, with downstream effects on retrieval. But mindfulness may play a direct role at the retrieval stage as well. For example, heightened attention during initial retrieval has been shown to enhance the probability of remembering the previously retrieved stimuli again in the future [80]. Research could help to specify whether mindfulness operates to enhance memory performance most effectively at encoding or at retrieval stages.

Finally, investigations of longer-term mindfulness training are needed to address the sustainability of its memory-enhancing effects. Relatedly, future research could also test the efficacy of mindfulness interventions to enhance episodic memory performance outcomes in educational and work settings to determine whether the effects observed here translate into *in vivo* contexts. Such contexts, and the computer technologies used in them, do not always provide optimal climates for focused attention and autonomously (including intrinsically) motivated behavior (e.g., [81]), and the present research suggests that mindfulness may be a valuable internal support for day-to-day memory performance.

## Supporting Information

S1 Text Training instructions, Studies 2 and 3. (DOCX)
