# Correction: Mindfulness Enhances Episodic Memory Performance: Evidence from a Multimethod Investigation

**DOI:** 10.1371/journal.pone.0160280

**Published:** 2016-07-25

**Authors:** Kirk Warren Brown, Robert J. Goodman, Richard M. Ryan, Bhikkhu Anālayo

In Table 4, row "Guess," column "*η*_*p*_^2^," the value is incorrectly listed as .31. The correct value is .02.

**Table 4:** Mean (and Standard Error) A*ꞌ* Values in the Remember-Know Task by Experimental Condition (Study 2).

A*ꞌ*	Mindfulness	Control	*F*	*p*	*η*_*p*_^2^
Remember	.91 (.02)	.84 (.02)	7.61	.007	.08
Know	.70 (.02)	.73 (.02)	1.21	.28	.01
Guess	.51 (.02)	.55 (.02)	.97	.33	.02

*Notes*. *N* = 93. A*ꞌ* values represent recognition accuracy of target stimuli.
