# Supplementary material for: Mindfulness Enhances Episodic Memory Performance: Evidence from a Multimethod Investigation
Source: PLoS One. 2016 Apr 26;11(4):e0153309. doi: 10.1371/journal.pone.0153309 (PMC4846034; doi:10.1371/journal.pone.0153309)
Supplement: S1 Text — (DOCX) [file pone.0153309.s001.docx]

**Training Instructions (Study 2)**

**Pre-learning phase of Remember-Know task**

Following participants’ completion of the initial battery of questionnaires and reading of the basic study instructions, all participants were told in a gentle, relaxed, but firm tone:

*Now that everyone has had the opportunity to read a little more about the study and has some understanding of what to expect, I’d like to take a few moments to go over what this is all about, and explain the nature of the activities we will engaging in in more detail.*

*We are interested how modern approaches to learning might have a broader positive impact on the quality of people’s day-to-day lives than previously expected. Taking an active, engaged approach in our lives may help usher in and deepen our connection with ourselves, with others, and with the world around us—both inside and outside of the classroom.*

*Here is how the remainder of the study will unfold. Everyone in this study listens to two audio recordings over headphones with their eyes closed. The audio recordings are identical for everyone. However, there are two conditions to this study. In one condition, we ask people to give their full attention to what is being said during the audio recordings, and to connect as best as you can with our experience of the content. In a second condition, people listen to the same audio recordings, but we distract them from being able to devote their full attention to them. Everyone in this experimental session has been randomly assigned to the active engagement condition. Let me explain what that means in a bit more detail and outline what you will be doing.*

*We ask that you devote your full attention to what is being said during the recordings, and genuinely enter into the experience of active participation. The audio recordings are not trying to trick you in any way, and there is no need to try to memorize what is being said during the recordings. Just be comfortable, relax, and actively listen. You will not be tested on anything that is said during the audio recordings. We simply ask that you sit up straight in your chair, gently rest your hands in your lap, and make your best effort to give your full attention to the recording, to be fully engaged anything it may ask you to do or think about. At times you may feel weird or awkward while engaging with the recordings. This is completely normal. Just completely engage with the recording for the short duration it is playing.*

*When the audio recording is over and you open your eyes, you will immediately start viewing a sequence of pictures on the computer screen. Your task is to sustain the active, engaged orientation you had while listening to the audio recordings while looking at each picture. Later on in the study, you will be asked to complete a kind of recognition exercise that will include the images that appear on the screen after you listen to the audio recording.*

*Please put the headphones on, enter the password, and do your best to devote your full attention to what is being said. Try to deeply connect with the experience.*

**Audio recording-delivered mindfulness condition instructions (randomized to receive male or female-voiced instruction first)**

Male voice (adapted from [1])

Female voice (adapted from [2])

**Audio recording-delivered active control condition instructions (randomized to receive male or female-voiced instruction first)**

Male voice (adapted from [3])

Female voice (adapted from [4])

**Pre-test phase of Remember-Know task**

*Now we are ready to move on to the second phase of the study. During this phase you will listen to another audio recording with your eyes closed, just as before. Do your best to fully experience the audio recording and actively participate. After the audio recording has ended, your memory for the pictures you viewed will be tested. Let me tell you a little more about how that will go.*

*After engaging with the audio recording, you will open your eyes and immediately begin a recognition exercise for the pictures you just viewed. During this part of the study, pictures will be displayed in the center of the screen-- just like before. For each picture, please respond "old" if the image was presented during the earlier part of the study. Respond "new" if the picture is new, and was not displayed during an earlier part of the study.*

*Again, do your best to use what you learned from the audio recording when engaging in this recall task.*

*If you respond "old" to a picture -- meaning you recognize seeing it earlier -- you will be asked what type of experience you are had when you recognized it.*

*The first type of recognition experience is called “Remembering”. A "Remember" response means that your recognition of the object is accompanied by a conscious, vivid recollection of your past experience viewing the picture. In other words, you are consciously aware again of something you experienced at the time the picture was first presented. That is, you re-live the experience of when you viewed the image in the past.*

*The second type of recognition experience is called “Knowing”. A "Know" response means that you recognize seeing the picture before, and you have strong feelings that the picture was presented before, but do not have the conscious experience of seeing the picture in the past when it was initially presented.*

*The third type of recognition experience is called “Guessing.” A "Guess" response means that you are simply guessing that the picture was displayed before, but you have no strong feelings that it was presented, nor any conscious recollection of your past experience viewing the image.*

*Does anyone have any questions about how to make these judgments?*

*Let try a few practice trials to make sure everyone understands the procedure.*

*Please sit up straight in your chair and keep both of your hands on the keyboard for the duration of this practice session. When you are ready, you can begin by entering the password.*

**Audio recording-delivered mindfulness condition instructions (received male- or female-voiced instruction that was not received in pre-learning phase)**

Male voice (as above)

Female voice (as above)

**Audio recording-delivered active control condition instructions (received male- or female-voiced instruction that was not received in pre-learning phase)**

Male voice (as above)

Female voice (as above)

**Training Instructions (Study 3)**

Following completion of the first reading measures, all participants were told:

“Before beginning the second reading, we want to make sure everyone is in the same mental state. So you’ll be listening to a tape to help get you to the same starting point as everyone else. You’ll be listening to this tape for about 10 minutes. Please keep the headphones on until the instructions tell you to go to the next part of the study.”

Distraction condition participants were in addition told:

“By the way, I should mention that this tape is not very good quality, unfortunately.”

**Audio recording-delivered mindfulness and distraction condition instruction**

As per Study 2 mindfulness instruction, male voice.

**References**

1. Segal, Z. V., Williams, J. M., & Teasdale, J. D. (2002). *Mindfulness-based cognitive therapy for depression: A new approach to preventing relapse.* New York: Guilford.
2. Brach, T. (2012). <https://www.tarabrach.com/guided-meditations/>
3. Covey, S. R. (1989). *The 7 habits of highly effective people.* New York: Simon and Schuster.
4. Arch, J. J., Brown, K. W., Dean, D. J., Landy, L. N., Brown, K. D., & Laudenslager, M. L. (2014). Self-compassion training modulates alpha-amylase, heart rate variability, and subjective responses to social evaluative threat in women. *Psychoneuroendocrinology*, *42*, 49-58.
